# High-Density Mapping of Triple Rust Resistance in Barley Using DArT-Seq Markers

**DOI:** 10.3389/fpls.2019.00467

**Published:** 2019-04-26

**Authors:** Peter M. Dracatos, Rouja Haghdoust, Ravi P. Singh, Julio Huerta Espino, Charles W. Barnes, Kerrie Forrest, Matthew Hayden, Rients E. Niks, Robert F. Park, Davinder Singh

**Affiliations:** ^1^Plant Breeding Institute Cobbitty, Sydney Institute of Agriculture, The University of Sydney, Sydney, NSW, Australia; ^2^International Maize and Wheat Improvement Center, Texcoco, Mexico; ^3^Campo Experimental Valle de México, INIFAP, Chapingo, Mexico; ^4^Instituto Nacional de Investigaciones Agropecuarias (INIAP), Quito, Ecuador; ^5^Agriculture Victoria, AgriBio, Centre for AgriBioscience, La Trobe University, Melbourne, VIC, Australia; ^6^Plant Breeding, Wageningen University & Research, Wageningen, Netherlands

**Keywords:** high-density linkage map, DArT-Seq markers, rust resistance, QTL, barley

## Abstract

The recent availability of an assembled and annotated genome reference sequence for the diploid crop barley (*Hordeum vulgare* L.) provides new opportunities to study the genetic basis of agronomically important traits such as resistance to stripe [*Puccinia striiformis* f. sp. *hordei* (*Psh*)], leaf [*P. hordei* (*Ph*)], and stem [*P. graminis* f. sp. *tritici* (*Pgt*)] rust diseases. The European barley cultivar Pompadour is known to possess high levels of resistance to leaf rust, predominantly due to adult plant resistance (APR) gene *Rph20*. We developed a barley recombinant inbred line (RIL) population from a cross between Pompadour and the leaf rust and stripe rust susceptible selection Biosaline-19 (B-19), and genotyped this population using DArT-Seq genotyping by sequencing (GBS) markers. In the current study, we produced a high-density linkage map comprising 8,610 (SNP and *in silico*) markers spanning 5957.6 cM, with the aim of mapping loci for resistance to leaf rust, stem rust, and stripe rust. The RIL population was phenotyped in the field with *Psh* (Mexico and Ecuador) and *Ph* (Australia) and in the greenhouse at the seedling stage with Australian *Ph* and *Pgt* races, and at Wageningen University with a European variant of *Psh* race 24 (*PshWUR*). For *Psh*, we identified a consistent field QTL on chromosome 2H across all South American field sites and years. Two complementary resistance genes were mapped to chromosomes 1H and 4H at the seedling stage in response to *PshWUR*, likely to be the loci *rpsEm1* and *rpsEm2* previously reported from the cultivar Emir from which Pompadour was bred. For leaf rust, we determined that *Rph20* in addition to two minor-effect QTL on 1H and 3H were effective at the seedling stage, whilst seedling resistance to stem rust was due to QTL on chromosomes 3H and 7H conferred by Pompadour and B-19, respectively.

## Introduction

Plant pathogens of the *Puccinia* genus are some of the most feared and damaging diseases of cereal crops (Dean et al., 2012). Since Biblical times, cereal rust pathogens have plagued farmer’s fields causing significant yield losses and in severe cases crop failure and famine (Kislev, 1982). Barley is the fourth most important cereal crop in the world and is mainly used for malt production, animal feed and, in some regions, human consumption. Three main rust diseases currently threaten barley production: wheat stem rust, barley leaf rust and barley stripe rust, caused by *Puccinia graminis* f. sp. *tritici* (*Pgt*), *P. hordei* (*Ph*), and *P. striiformis* f. sp. *hordei* (*Psh*), with the disease abbreviated hereafter as WSR, BLR, and BYR, respectively. All three rust diseases affect malting quality through reductions in kernel plumpness, kernel weight, and germination, resulting in economic losses to producers as premiums are paid for malting grade barley (Roelfs, 1978; Dill-Macky et al., 1990; Roelfs et al., 1992; Steffenson, 1992).

BYR, caused by *Psh*, is usually a problem in cooler, wetter climates that often prevail at higher altitudes, and it is therefore renowned as a cold-temperature rust disease. Despite widespread global crop losses *Psh* has not colonized all barley growing regions, predominantly due to geographic isolation (Dantuma, 1964; Macer and Van den Driessche, 1966; Nover and Scholz, 1969; Chen et al., 1995; Chen, 2007). While *Psh* has not been detected in Australia, offshore testing of Australian barley cultivars in greenhouse seedling tests or in field adult plant tests suggest between 60 and 70% are susceptible to this pathogen (Dracatos P.M. et al., 2015; Wellings, 2007). This implies a *Psh* incursion for Australia poses a significant threat to the Australian barley industry. In contrast, leaf rust (BLR) occurs globally and is frequently detected on a seasonal basis and can cause up to 60% yield losses in susceptible varieties (Park et al., 2015). The *Ph* rust fungus has systematically evolved virulence for widely deployed leaf rust resistance genes, typically via stepwise mutation, or in regions where the alternate host (*Ornithogalum umbellatum*) is present via sexual recombination (Wallwork et al., 1992). Despite their comparatively infrequent occurrence, epidemics of WSR usually have devastating effects for both wheat and barley production (Park, 2007). Due to the involvement of the same causal pathogen as wheat (*Pgt*), early sown wheat and Triticale crops threaten late-sown barley. Furthermore, studies suggest that globally >95% of barley accessions are susceptible to the widely publicized virulent races of *Pgt* derived from Eastern Africa such as the Ug99 lineage (TTKSK) (Steffenson et al., 2017).

Genetic resistance is the most environmental friendly and economically efficient control method to reduce yield losses due to rust diseases. In contrast to wheat, where more than 80 cataloged stripe rust resistance genes exist, fewer stripe rust resistance genes have been characterized for barley. Despite this, numerous genetic studies have been performed to characterize the inheritance of stripe rust resistance in barley, especially that present in the 12 international standard differential barley genotypes (Nover and Scholz, 1969; Chen and Line, 1992). Diverse inheritance patterns mainly involving recessive resistance genes were reported by Chen and Line (1999) among the 12 international standard differential barley genotypes. They observed varying inheritance patterns including: two complementary recessive genes (Emir, Varunda, and Trumpf), two independent recessive genes (Trumpf), and single recessive (BBA 2890, Grannelose Zweizeilige, I5 and PI 548708) or both recessive and dominant genes (Abyssinian 14 and Stauffers Obersulzer). A similar situation also exists for stem rust, where there is a lack of available resistance that can be effectively deployed in barley breeding programs. The cloned *rpg4/Rpg5* gene complex is the only known resistance effective against the Ug99 lineage, although virulent races exist within North America. More recently, other resistances have been identified and characterized (Steffenson et al., 2017). In a very recent study, WSR adult plant resistance (APR) genes *Rpg2* and *Rpg3* were mapped in barley on chromosomes 2H and 5H, respectively (Case et al., 2018). Recent WSR epidemics in Sicily (Patpour et al., 2015) and Germany (Olivera Firpo et al., 2017) have highlighted the importance of diversifying resistance in barley germplasm, and also emphasize that Ug99 is not the only threat to cereal production.

In contrast, BLR resistance in barley is widely available, including 26 designated resistance genes and numerous QTLs for partial resistance (Qi et al., 1998, 1999; Park et al., 2015; Kavanagh et al., 2017; Yu et al., 2018). As previously mentioned, there are numerous examples of resistance gene breakdown (mainly those with major phenotypic effect) due to dynamic and rapid evolution in prevailing pathogen populations. Only a few leaf rust resistance genes have remained durable across different regions/environments, one of which is the APR gene *Rph20*. *Rph20* was first identified as a QTL (*Rphq4*) in the Dutch barley cultivar Vada (Qi et al., 1998) and was later characterized in Pompadour (Golegaonkar et al., 2010; Liu et al., 2011) and the Australian cultivar Flagship (Hickey et al., 2011) where it was mapped to chromosome 5HS (Hickey et al., 2011; Liu et al., 2011). *Rph20* was found to be present at high frequency in European barley germplasm and expressed as early as the third leaf stage (Wang et al., 2010; Singh et al., 2013). However, in some accessions with very high levels of APR, the resistance was hypothesized to be due to *Rph20* and the presence of a 2nd or 3rd genetic component (Golegaonkar et al., 2010; Wang et al., 2010; Singh et al., 2013; Rothwell et al., 2019).

Recent genomic advancements in barley have improved the ability to develop physical scaffolds and utilize sequence information for marker development. Nevertheless, whole genome sequencing of populations and/or multiple accessions for non-model crop species with large genomes such as barley is still relatively expensive. Genotyping-by-sequencing (GBS) has been used as an alternative to whole genome sequencing due to: 1/lower cost, 2/generation of high quality genetic markers, and 3/suitability of the markers for genomic prediction/selection. DArT-Seq^TM^ technology combines the DArT array hybridization complexity reduction method (Wenzl et al., 2004) with next generation sequencing and can be optimized for any species. DArT-Seq has been used across numerous crop species for genetic diversity analysis (Baloch et al., 2017), genome-wide association studies (GWAS) (Singh et al., 2018; Visioni et al., 2018) and QTL mapping (Haghdoust et al., 2018). We have previously reported the utilization of DArT-Seq to map leaf rust (Dracatos et al., 2014; Singh et al., 2017, 2018) and stripe rust (Dracatos P.M. et al., 2015; Haghdoust et al., 2018) resistance in barley using marker-trait association and QTL mapping. In these studies, both the genetic and physical position of each marker was determined based on the Bowman consensus map and Morex physical reference assembly, respectively. To overcome possible differences in gene/marker order between the parents of the mapping population with the reference, and to improve the accuracy of mapping genes of interest, the use of trait-specific genetic maps is the preferred approach. In this study, we constructed a high-density linkage map using DArT-Seq markers for the Pompadour x Biosaline-19 (P/B-19) RIL population and precisely mapped QTLs for resistance to stripe, leaf and stem rust across different developmental stages and environments.

## Materials and Methods

### Plant Material

To study the inheritance of rust resistance at the seedling and adult plant stage, we used the F_9_-derived recombinant inbred line (RIL) Pompadour x Biosaline-19 (P/B-19) mapping population (98 RILs) developed at the Plant Breeding Institute, University of Sydney as described by Haghdoust et al. (2018). B-19 is widely susceptible to numerous *P. striiformis* formae speciales including *Psh*, as well as *Ph* at all developmental stages. In contrast, Pompadour is a French two-rowed feed spring barley that was previously determined to carry leaf rust resistance due mainly to the presence of *Rph20* (Golegaonkar et al., 2010; Liu et al., 2011) but is also resistance to stem and stripe rust.

### Greenhouse Inoculation and Phenotypic Analysis for Stripe Rust Resistance

Seedlings were grown and maintained in plant boxes as described in Niks et al. (2015) with the following exceptions. Following inoculation, the plant boxes were transferred to a dark dew chamber overnight, at 10°C. The rust susceptible B-19 parent and Dutch cultivar Vada were included in every tray along with the resistant parent Pompadour. Inoculation took place in a settling tower as described by Eyal et al. (1968) using a 1:12 mixture of urediniospores:Lycopodium spores with 3 mg of urediniospore being used to inoculate each tray. One isolate, Wageningen-derived *Psh* race 24 (*PshWUR*), was tested in two consecutive experiments. The responses of 10 barley genotypes at the seedling stage determined that *PshWUR* was virulent on Topper, Astrix, Atem, Berac and the susceptible control Vada, but avirulent on Agio, Bigo, Emir, Mazurka, Hiproly, Abed Binder, and Trumpf. Phenotypes were scored on a 0–4 scale, where those RILs with infection types (ITs) equal or greater than 3 were deemed susceptible and ITs 0–2 were deemed resistant.

### Phenotyping for BLR and WSR Resistance

Both seedling and field screening was performed as described by Singh et al. (2017). Only one *Ph* pathotype 5457 P+ (virulent on *Rph1*, *Rph2*, *Rph3*, *Rph4*, *Rph6*, *Rph9*, *Rph10*, *Rph12*, and *Rph19*) was used to inoculate the mapping population in the greenhouse at the seedling stage and in the field over two different seasons (2016 and 2 replicates in 2018) at Plant Breeding Institute, Cobbitty, NSW, Australia. Seedlings of the P/B-19 mapping population were inoculated and ITs recorded 10 and 12 days post inoculation using the 0–4 scale (Park and Karakousis, 2002), while the P/B-19 RILs were assessed in the field when the susceptible spreader control “Gus” reached a rating of 100S using a modified Cobb scale (Peterson et al., 1948) and 1–9 McNeal scale. The inoculation and disease assessment for stem rust resistance in the greenhouse at the seedling stage was performed using *Pgt* race 98-1,2,3,5,6 as described by Dracatos P. et al. (2015).

### Assessments of BYR Resistance in Ecuador

The P/B-19 RIL population was assessed for response to BYR in 2017 in Ecuador at the Instituto Nacional de Investigaciones Agropecuarias – INIAP station near Quito. The RILs were sown in 1 m × 1 m blocked groups and each block contained six lines in 1-m-long rows, spaced equally within the 1 m block. Each block was spaced roughly 30 cm apart. Five blocks were sown between susceptible spreader rows containing equal proportions of Shayari 89, Shayari 2000, and other local susceptible barley lines (including B-19). Spreader rows were not artificially inoculated. Each row of five blocks was spaced 1 m from the next row of blocks. Two evaluators scored each RIL simultaneously. The RILs were evaluated 70, 81, and 98 days later for disease severity according to the modified Cobb scale (Peterson et al., 1948).

### Phenotyping for BYR at Toluca, Mexico

The phenotyping for resistance to BYR was conducted at CIMMYT’s research station near Toluca (2640 mask, 18°N latitude), Mexico during the summer season coinciding with cooler conditions and high rainfall. The field plots of lines consisted of 1 m paired rows sown with about 60–80 seeds on top of 0.8 m wide raised beds. A susceptible spreader, variety Apizaco 36, was sown around the experimental field and as hills in the middle of the 0.5 m wide alleys on one side of each plot to allow uniform disease development. Greenhouse increased fresh urediniospores of Mexican variant of race 24 (*PshMEX-1*) of *Psh* were suspended in Soltrol 170 oil and sprayed onto about 1 month old spreaders. The differential response on 10 barley genotypes at the seedling stage determined that *PshMEX-1* was virulent on Topper, Cambrinus, Mazurka, Varunda, Emir, Heils Franken, Abed Binder, and Trumpf, but avirulent on Bigo, I5 and the bread wheat cultivar Morocco (Sandoval-Islas et al., 1998).

BYR severity was recorded twice according to the modified Cobb scale (Peterson et al., 1948), when the severity on the susceptible control Kaputar reached approximately 60 and 100%, respectively. In addition, the host responses to infection were also determined according to Roelfs et al. (1992).

### DArT-Seq Marker Genotyping and Genetic Map Construction

Genotyping-by-sequencing (GBS) data was generated using the DArT-Seq platform (DArT PL, Canberra, NSW, Australia) as described on the company website^1^. Marker sequences were aligned against the Morex barley genome assembly (Mascher et al., 2017) using the sequence aligner Nuclear (Gydle Inc., Bioinformatics Service, Quebec City, QC, Canada) with three mismatches allowed.

Both dominant and co-dominant markers with at least 70% call rate were considered for map construction. A preliminary map was constructed in R/ASMap (Taylor and Butler, 2017) using the Kosambi mapping function at LOD 6. R/ASMap was used to identify and remove redundant markers, rectify markers with switched alleles, remove duplicate samples and lines with high (≥30%) missing data, a high number of crossovers, or high (≥10%) heterozygosity, the latter also being an indicator of a mixed sample. Markers with segregation distortion (*p*-value <0.01 calculated from a χ^2^ test) that had inconsistent ordering compared to the Morex genome assembly and unlinked markers were excluded. Distorted markers were not automatically discarded as some linkage groups showed regions with high density of low-level distortion, presumably with biological significance. Following marker and sample filtering, the map was reordered in R/ASMap at LOD 8.

### QTL Mapping in the P/B-19 RIL Population

We used the same genotypic dataset previously described in Haghdoust et al. (2018). A high-density linkage map was constructed comprising 8,610 markers (SNPs and *in silico* DArT-Seq markers). In contrast to Haghdoust et al. (2018), we selected markers every 10–15 cM based on genetic positions from the newly constructed linkage map spanning >5,000 cM. IT data from the greenhouse for leaf, stem, and stripe rust were converted to binary scores and mapped using composite interval mapping (CIM). Field-based data for leaf and stripe rust from each disease nursery were mapped using quantitative disease measurements (either Cobb 0–100 or McNeal 1–9). Additionally, phenotypic data for seedling resistance to the barley grass stripe rust pathogen (*P. striiformis* f. sp. *pseudo-hordei*) from Haghdoust et al. (2018) was also used for QTL analysis using the new genetic map with the aim of comparing the position of stripe rust resistance QTLs. QTL regions associated with resistance to different *Psh* isolates in the P/B-19 RIL population were considered to be the same if there was overlap between their LOD-1 support intervals.

## Results

### Greenhouse and Field *Psh* Inoculations and Inheritance of Resistance

**Figure 1:**
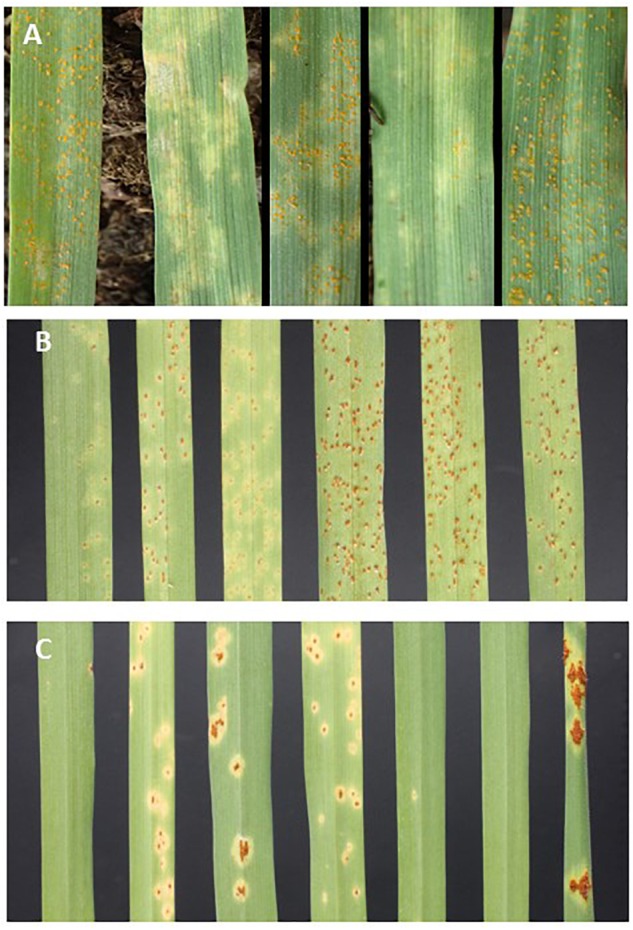
Seedling leaves infected with **(A)** *PshWUR* (L to R) B-19, Pompadour, RIL38, RIL39, RIL42 14 days post inoculation (dpi), **(B)** *P. hordei* pathotype 5457 P+ (L to R) Pompadour, B-19, RIL72, RIL52, RIL3, Estate (*Rph3*). **(C)** *P. graminis* f. sp. *tritici* pathotype 98-1,2,3,5,6 (L to R) Pompadour, B-19, RIL64, RIL2, RIL63, RIL7 and the susceptible wheat accession Morocco 12 dpi.

In greenhouse phenotypic assessments at the seedling stage with the *PshWUR* isolate, Pompadour was highly resistant (IT = “;C”) and B-19 was very susceptible (IT = “4”) based on the “0–4” IT scale described in Haghdoust et al. (2018). To determine the genetic basis of resistance in the Pompadour parent, we screened the P/B-19 RIL population at the seedling stage in the greenhouse in two separate experimental replicates (Figure 1A). Across both experimental replicates, >68% of the RILs were highly susceptible (IT = “3+” to “4”). Three phenotypic responses were observed in the P/B-19 RIL population, *viz*. highly resistant similar to Pompadour, intermediate (restricted pustules) and fully compatible similar to the susceptible parent B-19 (Figure 1A). Chi Squared analysis suggested that the segregation ratio best fitted a 1R : 3S ratio (χ^2^ = 0.154 at 1 d.f.; *p* = 0.695), supporting the presence of two complementary resistance genes conferring seedling resistance to *PshWUR*.

**Figure 2:**
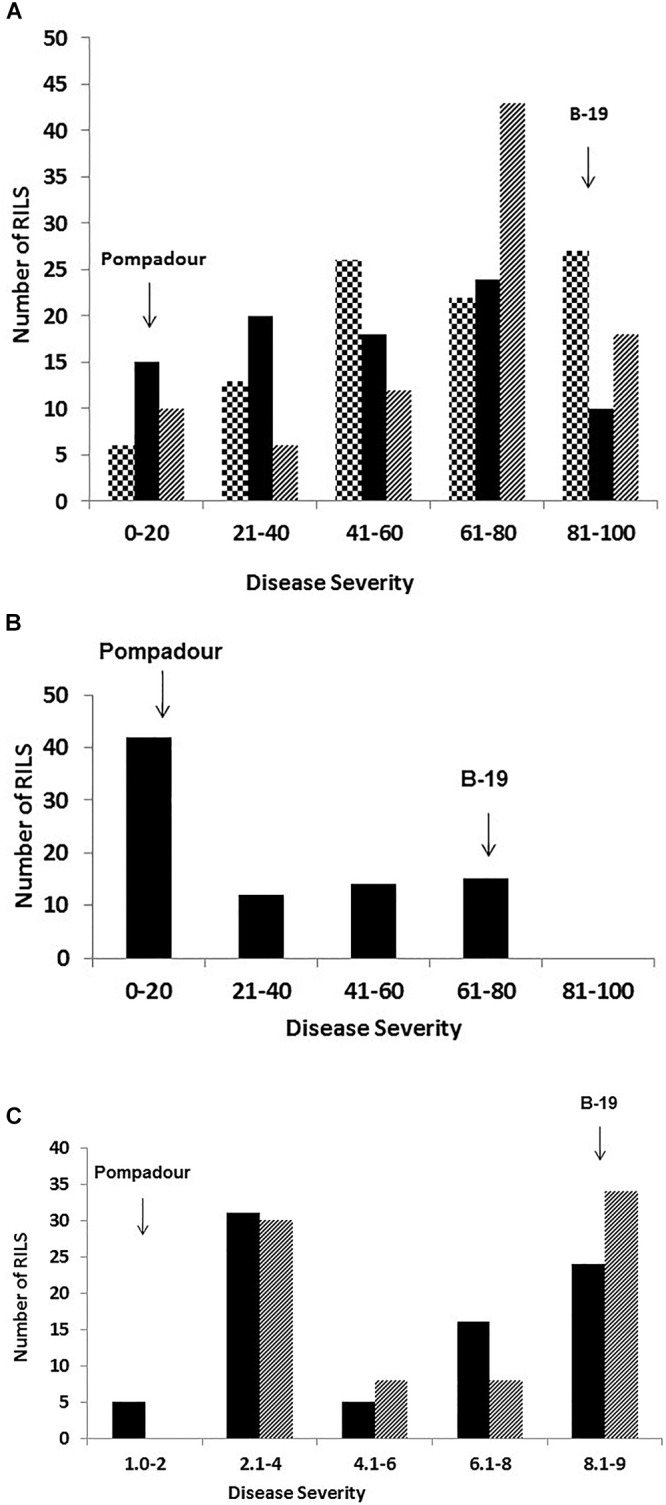
Frequency distribution of disease severity of 98 Pompadour/Biosaline-19 RILs at the adult plant stage in response to **(A)** *Puccinia striiformis* f. sp. *hordei* in Toluca Mexico in 2015 (large checker board) and in 2016 (black), and in Ecuador in 2017 (downward diagonal) using a modified Cobb scale; and *P. hordei* in the field at Cobbitty, Australia **(B)** in 2016 using a modified Cobb scale, and **(C)** two different ecperimental replicated in 2018 using a 1-9 McNeal scale, respectively. Disease severity values for parents are indicated by arrows.

At the Ecuador field site, Pompadour and B-19 had a mean disease rating of 2.5 and 90% disease severity, respectively. Very similar results were observed in Mexico in 2015 and 2016 seasons. The frequency distribution histogram derived from field assessment data from Ecuador in 2017 was clearly skewed toward susceptibility, whilst data from Toluca in 2015 and 2016 seasons were also skewed toward susceptibility but were a closer fit to a normal distribution, respectively (Figure 2A).

### Inheritance of Resistance to *Ph* and *Pgt*

In response to *Ph* pathotype 5457 P+, Pompadour gave an intermediate response of “;1+C” at the seedling stage, and was highly resistant in the field (1R). In contrast, the B-19 parent was highly susceptible (“3+” and 90S, respectively) (Figure 1B). Barley leaf rust resistance in the RIL population segregated bimodally (1R : 1S) and was simply inherited at both the seedling (χ^2^ = 0.013 at 1 d.f.; *p* = 0.909) and adult plant stages (2016 and 2018) (Figure 2B,C), with the segregation pattern best fitting monogenic inheritance which was highly correlated to the presence of the *Rph20* marker *bPb-0837*. In contrast to the phenotypic response to both leaf rust and stripe rust, both parental genotypes were resistant (Pompadour IT = “0;” and B-19 IT “;12C”) to *Pgt* race 98-1,2,3,5,6 at the seedling stage in the greenhouse. Pompadour was highly resistant and B-19 showed an intermediate response as shown in Figure 1C. The segregation for resistance in the P/B-19 mapping population best fitted a two-gene model (χ^2^ = 0.974 at 1 d.f.; *p* = 0.324), suggesting the involvement of resistance from both parents.

### Genetic Map Construction

A total of 18,062 DArT-Seq markers showing polymorphism in the P/B-19 RIL population were considered for genetic map construction. Linkage map construction was performed using ASmap LOD8 following marker curation. Markers that were redundant, had significant segregation distortion (*p* < 0.01) and/or low call rate (<70%), were removed as were duplicated samples, or those with missing data (>70%) (Supplementary Table S1). Following quality filtering, the final map consisted of 1,596 codominant SNP and 7,014 dominant DArT-Seq markers that spanned 5,957.6 cM across seven linkage groups corresponding to chromosomes 1H-7H (Supplementary Table S1). Approximately 50% of the markers could not be unambiguously assigned to a physical position in the 2017 Morex reference genome assembly; in 123 cases this was due to the presence of >2 map matches presumably due to paralogous gene families. In contrast, 4,220 markers were mapped to unique physical positions in the Morex genome. However, on numerous occasions the position determined in the P/B-19 linkage map did not correspond to that of the Morex reference. Numerous linkage blocks were identified during map construction, evidenced by the abundance of redundant markers. Nevertheless 4,220 markers could be mapped to unique physical positions in the Morex genome. Numerous regions in the linkage groups contained multiple redundant markers. Twenty-three such regions had >50 co-segregating markers including a region on chromosome 3H that had 233 redundant markers (Supplementary Table S1). This could be indicative of regions of the genome with repressed recombination or possibly an introgression from a wild *Hordeum* spp.

### QTL Analysis for Rust Resistance

**Figure 3:**
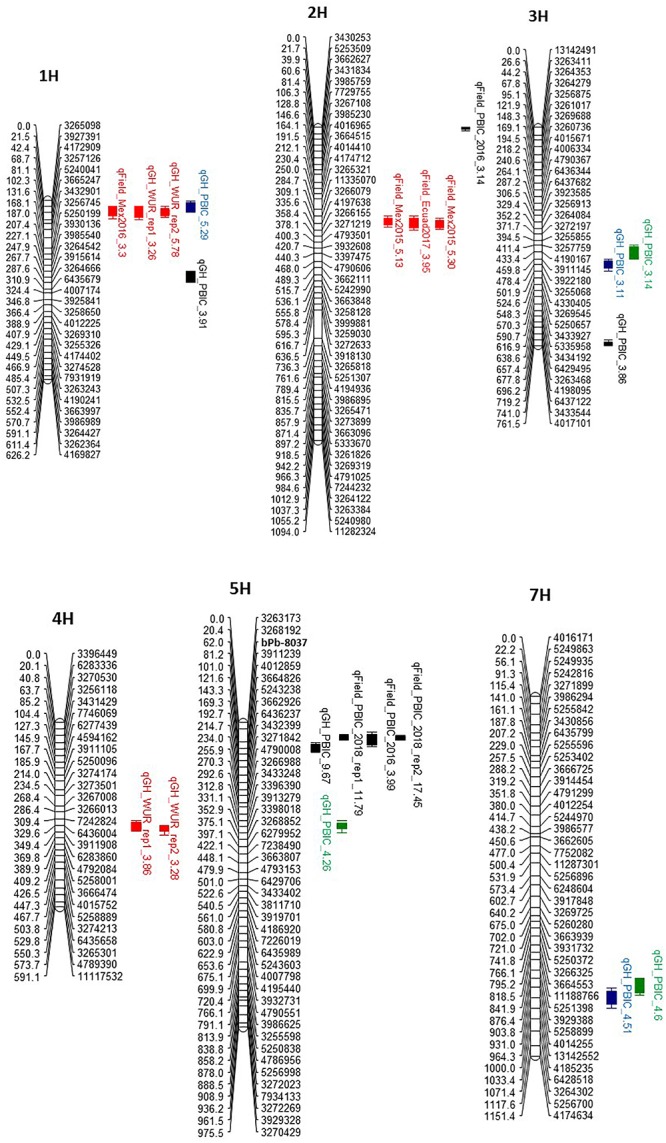
Quantitative trait loci (QTL) for resistance on chromosomes 1H, 2H, 3H, 4H, 5H, and 7H in the Pompadour/Biosaline-19 RIL population to *Puccinia striiformis* f. sp. *hordei* (red), *P. striiformis* f. sp. *pseudo-hordei*) (blue), *P. graminis* f. *sp. tritici* (green), and *P. hordei* (black). The name on QTL bars has three components: the provisional name of the gene at the QTL, location/isolate and the logarithm of odds (LOD) value recorded for the QTL.

The high-density linkage map was used to precisely map QTL for rust resistance segregating in the P/B-19 mapping population. A total of 10 QTLs (exceeding the LOD threshold of 3) were mapped with distinct chromosomal locations for resistance to the four different rust diseases (leaf, stem, and stripe rust caused by both barley and barley grass-adapted formae speciales of *P. striiformis*) (Table 1). The LOD scores ranged from 3.11 to 17.45 for all QTLs and the percentage of variation explained by individual QTLs ranged from 11 to 36%, but was mostly lower than 20%, indicating that the mapped rust resistance at the seedling and adult plant stages were due to genes with both small and large effect (Table 1 and Figure 3).

**Table 1:** Summary of QTLs for rust resistance identified in the Pompadour × Biosaline-19 RIL population.

Trait	Location	Stage	Pathogen isolate	Chromosome	Peak LOD	Position (cM)
qField_Mex2015_3.30	Toluca, Mexico_2015	Adult	Psh Mex-1	1H	3.3	37.55
qField_Mex2015_5.13	Toluca, Mexico_2015	Adult	Psh Mex-1	2H	5.13	327.06
qField_Mex2016_5.30	Toluca, Mexico_2016	Adult	Psh Mex-1	2H	5.3	338.59
qField_Ecuad2017_3.95	Ecuador_2017	Adult	Field infection (mixed)	2H	3.95	335.59
qGH_WUR_rep1_3.26	WUR_greenhouse	Seedling	*PshWUR*	1H	3.26	42.37
qGH_WUR_rep1_3.86	WUR_greenhouse	Seedling	*PshWUR*	4H	3.86	329.64
qGH_WUR_rep2_5.78	WUR_greenhouse	Seedling	*PshWUR*	1H	5.78	43.37
qGH_WUR_rep2_3.28	WUR_greenhouse	Seedling	*PshWUR*	4H	3.28	339.64
qGH_PBIC_5.29	PBIC_greenhouse	Seedling	*Psph* isolate 9865566	1H	5.29	35.55
qGH_PBIC_3.14	PBIC_greenhouse	Seedling	*Psph* isolate 9865566	3H	3.14	472.8
qGH_PBIC_4.26	PBIC_greenhouse	Seedling	*Psph* isolate 9865566	5H	4.26	331.07
qGH_PBIC_4.51	PBIC_greenhouse	Seedling	*Psph* isolate 9865566	7H	4.51	967.33
qGH_PBIC_3.91	PBIC_greenhouse	Seedling	*P. hordei* 5457 P+	1H	3.91	272.71
qGH_PBIC_3.86	PBIC_greenhouse	Seedling	*P. hordei* 5457 P+	3H	3.86	761
qGH_PBIC_9.67	PBIC_greenhouse	Seedling	*P. hordei* 5457 P+	5H	9.67	79.04
qField_PBIC_2018_rep1_11.79	PBIC_Australia_2018_rep1	Adult	*P. hordei* 5457 P+	5H	11.79	49.39
qField_PBIC_2016_3.14	PBIC_Australia_2016	Adult	*P. hordei* 5457 P+	2H	3.14	0.01
qField_PBIC_2016_3.99	PBIC_Australia_2016	Adult	*P. hordei* 5457 P+	5H	3.99	48.9
qField_PBIC_2018_rep2_17.45	PBIC_Australia_2018_rep2	Adult	*P. hordei* 5457 P+	5H	17.45	49.39
qGH_PBIC_3.11	PBIC_greenhouse	Seedling	Pgt 98-1,2,4,5,6	3H	3.11	443.79
qGH_PBIC_4.6	PBIC_greenhouse	Seedling	Pgt 98-1,2,4,5,6	7H	4.6	933.03

For BYR resistance using the *PshWUR* isolate, the IT data from both seedling greenhouse experiments were highly correlated (*r* = 0.8) and distinct phenotypic classes (of resistant and susceptible) were easily distinguished and, therefore qualitative assessments were taken from all RILs for the purpose of QTL mapping. We identified two consistent QTLs for resistance at the seedling stage on chromosomes 1H and 4H, both contributed by Pompadour (Table 1 and Figure 3). For both experiments, the mapping resulted in identical LOD profiles (Supplementary Figure S1A). The infection level of the P/B-19 RILs to BYR at the adult plant stage at field sites in both Mexico and Ecuador was assessed quantitatively and the segregation was continuous but skewed toward susceptibility. In 2016 at Toluca, Mexico, the same 1H QTL was identified as in the two greenhouse seedling experiments using both *PshMex-1* and *PshWUR* isolates, respectively, while in 2015 (Toluca, Mexico) a signal with LOD 2.8. This QTL was not detected in 2017 from the Ecuador-derived dataset (Figure 3 and Supplementary Figure S1A). Consistent QTL on chromosome 2H were identified across all field environments, but was not identified in the greenhouse in response to the *PshWUR* isolate.

We also phenotyped the P/B-19 RIL population and mapped QTL for resistance to both leaf and stem rust. For leaf rust, we phenotyped the population in both the greenhouse under controlled conditions and in the field at Plant Breeding Institute Cobbitty over three successive seasons at the adult plant stage using the same *Ph* pathotype (5457 P+). We identified three QTLs for resistance on chromosomes 1H, 3H, and 5H at the seedling stage based on two highly correlated (*r* = 0.9) experimental replicates. From the field data, in all instances, we mapped QTL on the short arm of chromosome 5H corresponding to the position of *Rph20*, while an additional QTLs were also mapped only for the 2016 data on the short arm of chromosome 2H. The major-effect leaf rust QTLs identified on chromosome 5H are all in close proximity or co-locate with the *bPb-0837* marker that is associated with the *Rph20* resistance. The 1H leaf rust QTL was only effective at the seedling stage and was located near the centromere (Figure 3, Table 1, and Supplementary Figure S1B). For stem rust, two minor effect QTL (LOD score <5) for seedling resistance mapped on chromosomes 3H and 7H based on two experimental replicates (*r* = 0.7) (Figure 3, Supplementary Table S1, and Supplementary Figure S1C).

Co-location of QTLs against multiple rust pathogen species was identified on four different chromosomes, *viz*. 1H, 3H, 5H, and 7H. We remapped the phenotypic data reported in Haghdoust et al. (2018) for barley grass stripe rust resistance at the seedling stage using the genetic map created in the present study (Figure 3). The same QTL on chromosomes 1H, 3H, 5H, and 7H identified in Haghdoust et al. (2018) were also identified using the high-density genetic map. The chromosome 1H QTL co-located with the broadly effective QTL identified for BYR resistance, while the 3H and 7H QTLs co-located with both stem rust resistance loci mapped in this study (Figure 3).

## Discussion

The accuracy and speed taken to characterize disease resistance genes in plants has increased due to advancements in genomics, especially through cheaper sequencing technologies. GBS-derived complexity reduction methodologies such as DArT-Seq generate thousands of polymorphic markers that can be used to expand the size of linkage groups. We previously mapped both leaf rust (Dracatos et al., 2014; Kavanagh et al., 2017) and stripe rust (Dracatos P.M. et al., 2015) resistance in barley and developed a high-density linkage map in wheat to map both components of complementary stripe rust resistance (Dracatos et al., 2016) using the DArT-Seq marker platform. To enable precise mapping of QTLs for resistance to stripe rust, leaf rust and stem rust derived from the European malting barley cv. Pompadour, we constructed a linkage map for P/B-19 RIL population reported in Haghdoust et al. (2018) using 8,610 polymorphic DArT-Seq and SNP markers spanning >5,000 cM.

**Figure 4:**
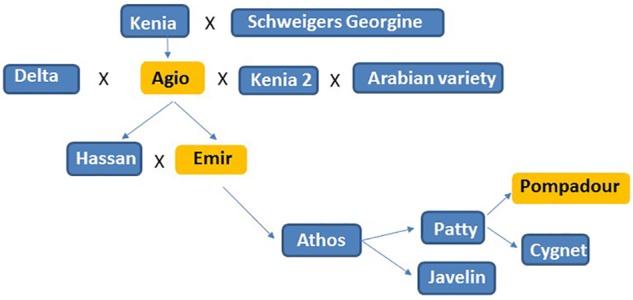
Pedigree analysis of the Pompadour parent, accessions in orange were resistant to *Psh* isolates *PshWUR* and *PshMex-1* used in this study.

The ability of cereal rust pathogen spores to migrate both within and between continents and hence adapt to new environmental conditions and host genotypes continue to provide challenges to cereal production (Brown and Hovmøller, 2002; Ali et al., 2014). In cases where phenotypic analysis has to be performed offshore at international field and greenhouses because a pathogen is not present, as is the case for *Psh* in Australia, using a genetic map with high marker density to resolve the location of the R gene/QTL in the first instance can enhance the efficiency of subsequent pre-emptive breeding efforts. Phenotyping and genetic analysis for BYR resistance in the greenhouse determined that Pompadour likely carries two complementary resistance genes effective at the seedling stage in response to inoculations with *PshWUR*. CIM analysis identified the presence of two QTLs for stripe rust resistance at the seedling stage on chromosome 1H and 4H in both greenhouse experiments. We included differentials Emir and Agio in our tests and determined both shared a similar disease response to *PshWUR* as the Pompadour parent. Pedigree analysis of Pompadour parent revealed that it was derived from Emir through Agio, suggesting that the observed seedling resistance is likely *rpsEm1* and *rpsEm2* genes (Figure 4). Further intercrossing and subsequent rust testing is required to truly confirm the complementary gene hypothesis, following the procedures used in wheat by Dracatos et al. (2016) to map the complementary stripe rust resistance genes *Yr73* and *Yr74*. We also assessed the P/B-19 mapping population at high altitude cooler regions in Ecuador and Mexico under field conditions over three successive seasons. The same 1H QTL was also mildly effective in Toluca, Mexico in 2016 (not significant in 2015, LOD = 2.78) at the adult plant stage in the field, suggesting it may be interactive with the 2H QTL. In contrast to the *PshWUR* isolate we used for mapping of seedling resistance, the isolate used in Toluca (*Mex-1*) was virulent with respect to the Emir (*rpsEm1* and *rpsEm2*) resistance. Resistance to *PshWUR* was previously mapped by Niks et al. (2015) on chromosome 4H in the Vada x L94 mapping population and was contributed by the L94 parent and believed to be derived from the Ethiopian accession Grannelose Zweizeilige. We did not identify the 4H QTL from the data from Mexico or Ecuador, suggesting it is either ineffective to the *Mex-1* isolate and Ecuadorian *Psh* population, or only effective at the seedling stage. A similar result was previously observed for the *Lr12/Lr31* wheat leaf rust complementary gene system where only *Lr12* conferred resistance in adult plants (Singh et al., 1999).

Stripe rust resistance genes have previously been reported in the telomeric regions of the short arm of chromosome 1H in barley were associated with the *Mla* locus that is renowned to harbor highly divergent R genes effective to powdery mildew in barley and in wheat stem rust (*Sr33* and *Sr50*) (Periyannan et al., 2013; Mago et al., 2015). Two recent studies have reported on a barley QTL in the same region conferring resistance to isolates of *P. striiformis* adapted to brome grass, barley-grass, and wheat, suggesting the presence of a cluster of resistance genes consistent with the *MLA* hypothesis. However, the presence of a broadly effective resistance gene cannot be ruled out (Niks et al., 2015; Kamino et al., 2016; Haghdoust et al., 2018). We re-mapped the 1H QTL for seedling and adult plant resistance to the barley grass adapted formae speciales of *P. striiformis* (which causes barley grass stripe rust – BGYR) reported by Haghdoust et al. (2018) in the P/B-19 using our high density linkage map and determined it also co-locates with the BYR seedling 1H QTL mapped in the present study, suggesting they are likely the same gene or tightly linked genes.

In this study, we mapped a broadly effective field-based resistance QTL on chromosome 2H. Further testing is required to determine if this resistance is also expressed at the seedling stage using the same Mexican and Ecuadorian isolates that occurred in the field. Field resistance to BYR has been mapped previously on chromosome 2H in four separate studies using QTL mapping [Shayari/Gardner (Toojinda et al., 2000) and BCD47/Baronesse (Vales et al., 2005) DH populations] and GWAS (Gutierrez et al., 2015; Visioni et al., 2018) approaches, respectively. Further genetic studies are required to determine whether these loci are distinct or the same. Interestingly, the 2H QTL identified in Gutierrez et al. (2015) was also effective under Ecuadorian field conditions, as was the 2H QTL derived from Baronesse (Vales et al., 2005) and may have also been derived from the European material included in their study likely suggesting the involvement of the same gene. The lack of common marker types between genetic maps prevents comparative mapping analysis. Most recently, Visioni et al. (2018) used the same marker platform (DArT-Seq) and performed a GWAS on a diverse collection of European, Australian and American (North and South) barley accessions to map stripe rust resistance in response to Indian *Psh* races. The Visioni et al. (2018) study also mapped the same QTL on chromosome 2H as reported here and in previous studies, suggesting that it might be a broadly effective and hence valuable source of stripe rust resistance providing broad-spectrum resistance under diverse environments.

Numerous barley accessions have been reported to carry the *Rph20* resistance, however, most are highly susceptible as seedlings using oil-based inoculation methods (Golegaonkar et al., 2010). Two previous studies using different *P. hordei* isolates and inoculation methods determined that (*Rphq4* – *Rph20*) was expressed from the 3rd leaf stage to maturity (Wang et al., 2010; Singh et al., 2013). In contrast, Wang et al. (2010) determined that a second QTL for partial resistance, *Rphq2* on chromosome 2HL, prolongs latent period at the seedling stage but has almost no effect on disease resistance in adult plants. We identified three QTLs for leaf rust resistance at the seedling stage, on chromosomes 1H, 3H, and 5H. The presence of the *bPb-0837* marker allele was strongly correlated with resistance in the P/B-19 RIL population, suggesting that the 5HS QTL detected is likely *Rph20*, however, the 1H QTL appears to be an uncharacterized QTL only expressed at the seedling stage. Our data suggests that in the Pompadour background, *Rph20* is expressed earlier than previously determined. Relative to the Australian cultivar Flagship, numerous European varieties including Pompadour are more resistant (near immune) to leaf rust under field conditions under high inoculum pressure (Golegaonkar et al., 2010). Whether this is due to additional QTL for partial resistance (as observed in Vada), allelic variation at the *Rph20* locus or variation in the pathogen population is yet to be determined. Our field data suggest that despite two experiment-specific minor effect QTLs, *Rph20* was consistently detected across all three replicates. Whether this indicates that the Pompadour *Rph20* allele confers stronger resistance or that other genetic components could not be effectively phenotyped due to environmental variation or inoculum pressure is unknown. Once *Rph20* is cloned, further studies of allelic variation and their phenotypic effect will be possible, and will be relevant to breeding programs and evolutionary studies.

Two cloned resistance genes (*Rpg1* and *rpg4/Rpg5*) have proved the most important to date for durable protection to stem rust in barley (Steffenson et al., 2017). Although *rpg4/Rpg5* is still widely effective to most global *Pgt* isolates, virulence for *Rpg1* is now common, and in some countries such as Australia, virulence is thought to be fixed. In this study, we were successful in mapping two QTLs for resistance to stem rust at the seedling stage on chromosomes 3H and 7H from greenhouse, derived from each of experiments using the parents of the P/B-19 RIL populations. A very recent study mapping QTL for resistance to stem rust in barley mapped both *Rpg2* and *Rpg3* on chromosomes 2H and 5H, respectively (Case et al., 2018). The same study identified multiple co-locating QTL, and a single QTL, on chromosomes 3H and 7H, respectively, in response to North American isolates of *Pgt* at the seedling stage (Case et al., 2018). Due to the lack of common marker types the relationship between these QTL is unknown. However, the 7H QTL identified in the present study appears to provide a high level of protection at the seedling stage and is potentially a valuable source of resistance for varietal improvement.

DArT-Seq is a high-throughput complexity reduction based GBS technology enhancing genetic capabilities including: high-density linkage map construction, GWAS, QTL mapping and genetic diversity analysis. In summary, we used DArT-Seq genotypic data to generate a high-density linkage map in the barley RIL population P/B-19 to precisely map resistance QTL to stripe, leaf, and stem rust pathogens. We determined Pompadour was a rich source of resistance to all three rust species. Further use of DArT-Seq marker sequence information is ongoing for marker development for the selection of triple rust resistance in barley. In the era of rapid gene cloning methodologies, future experiments to isolate the chromosome 2H and 4H QTLs (likely in a region of low recombination) should adopt a mutant sequencing approach as recently described for barley leaf rust resistance gene *Rph1* (Dracatos et al., 2019) to search for candidate gene(s) within the defined intervals confirmed in this study.

## Author Contributions

PD, RP, and DS conceptualized the work and designed the project. PD wrote the manuscript with contributions from RH, which was read, edited and approved by all co-authors. PD and RH performed the genetic and QTL analysis. PD, RH, DS, JHE, RN, RS, and CB performed the phenotypic analysis. MH and KF performed the genetic map construction.

## Conflict of Interest Statement

The authors declare that the research was conducted in the absence of any commercial or financial relationships that could be construed as a potential conflict of interest.

## Footnotes

1 https://www.diversityarrays.com/

## Supplementary Material

FIGURE S1 LOD trace files for **(A)** stripe [green and black = WUR greenhouse rep 1 and 2, dark blue, red, and black dash = Mexico and Ecuador field sites and light blue = *P. striiformis* f. sp. *pseudo-hordei*], **(B)**leaf [pink = greenhouse PBI Cobbitty, dark blue dash = Field_PBICobbitty_2018rep1, light blue = Field_PBICobbitty_2018rep2, black dashed = FieldPBICobbitty_2016] and **(C)** stem rust [red dashed = greenhouse PBI Cobbitty] resistance QTLs identified in the Pompadour × Biosaline-19 RIL population.

TABLE S1 Summarised marker information for the high-density linkage map constructed for the Pompadour/Biosaline-19 RIL population.
