# Supplementary figures and images for: High-Density Mapping of Triple Rust Resistance in Barley Using DArT-Seq Markers
Source: Front Plant Sci. 2019 Apr 26;10:467. doi: 10.3389/fpls.2019.00467 (PMC6498947; doi:10.3389/fpls.2019.00467)

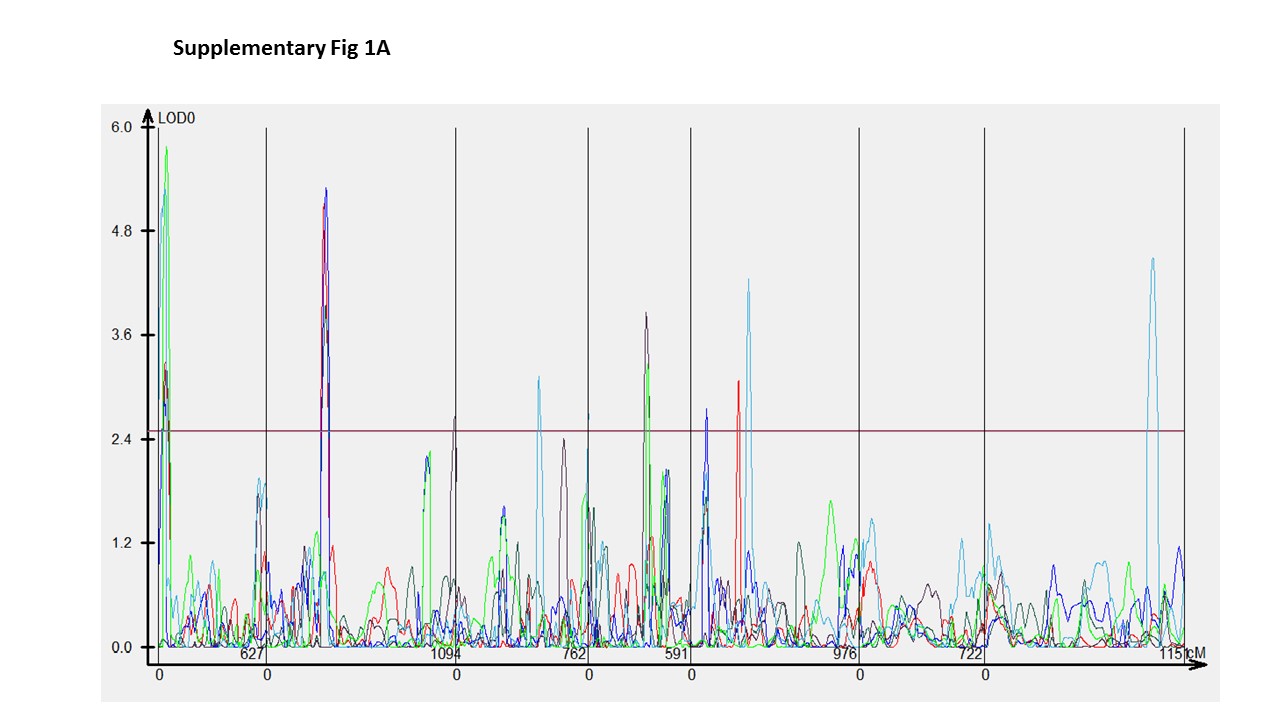

Supplement: FIGURE S1 — LOD trace files for (A) stripe [green and black = WUR greenhouse rep 1 and 2, dark blue, red, and black dash = Mexico and Ecuador field sites and light blue = P. striiformis f. sp. pseudo-hordei], (B)leaf [pink = greenhouse PBI Cobbitty, dark blue dash = Field_PBICobbitty_2018rep1, light blue = Field_PBICobbitty_2018rep2, black dashed = FieldPBICobbitty_2016] and (C) stem rust [red dashed = greenhouse PBI Cobbitty] resistance QTLs identified in the Pompadour × Biosaline-19 RIL population. [file Data_Sheet_1.zip › Image 1A.JPEG]

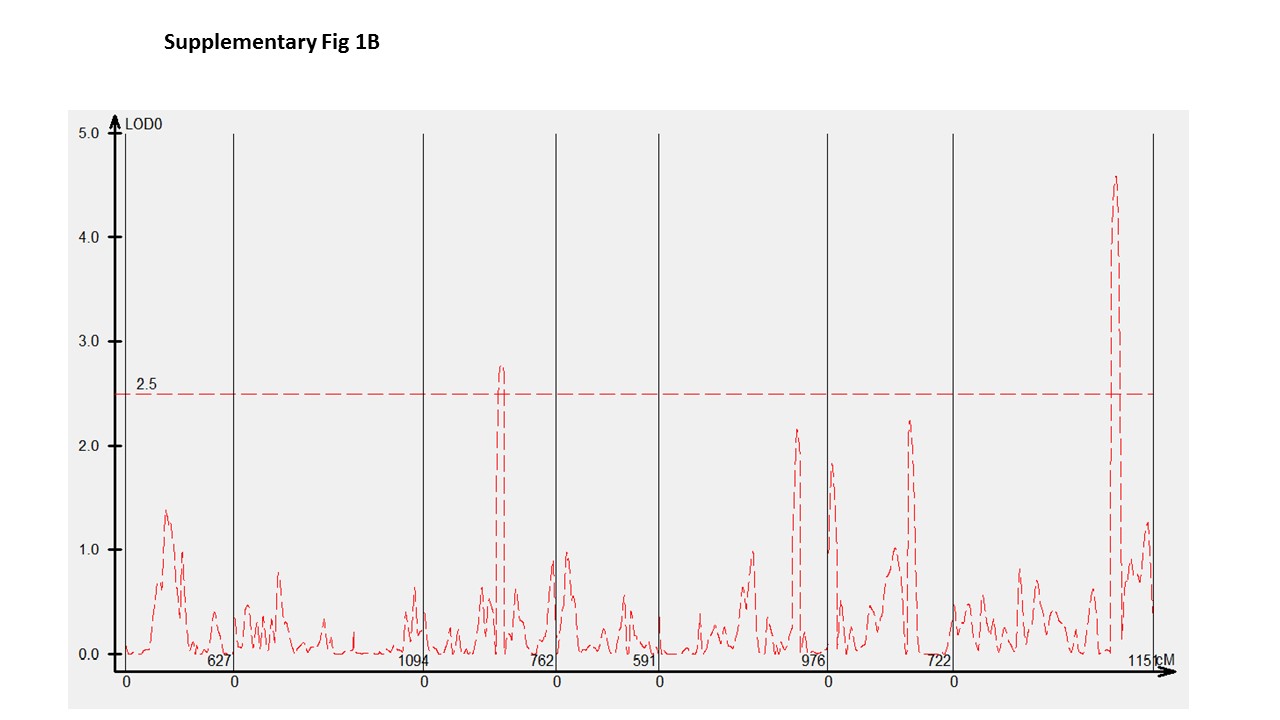

Supplement: FIGURE S1 — LOD trace files for (A) stripe [green and black = WUR greenhouse rep 1 and 2, dark blue, red, and black dash = Mexico and Ecuador field sites and light blue = P. striiformis f. sp. pseudo-hordei], (B)leaf [pink = greenhouse PBI Cobbitty, dark blue dash = Field_PBICobbitty_2018rep1, light blue = Field_PBICobbitty_2018rep2, black dashed = FieldPBICobbitty_2016] and (C) stem rust [red dashed = greenhouse PBI Cobbitty] resistance QTLs identified in the Pompadour × Biosaline-19 RIL population. [file Data_Sheet_1.zip › Image 1B.JPEG]

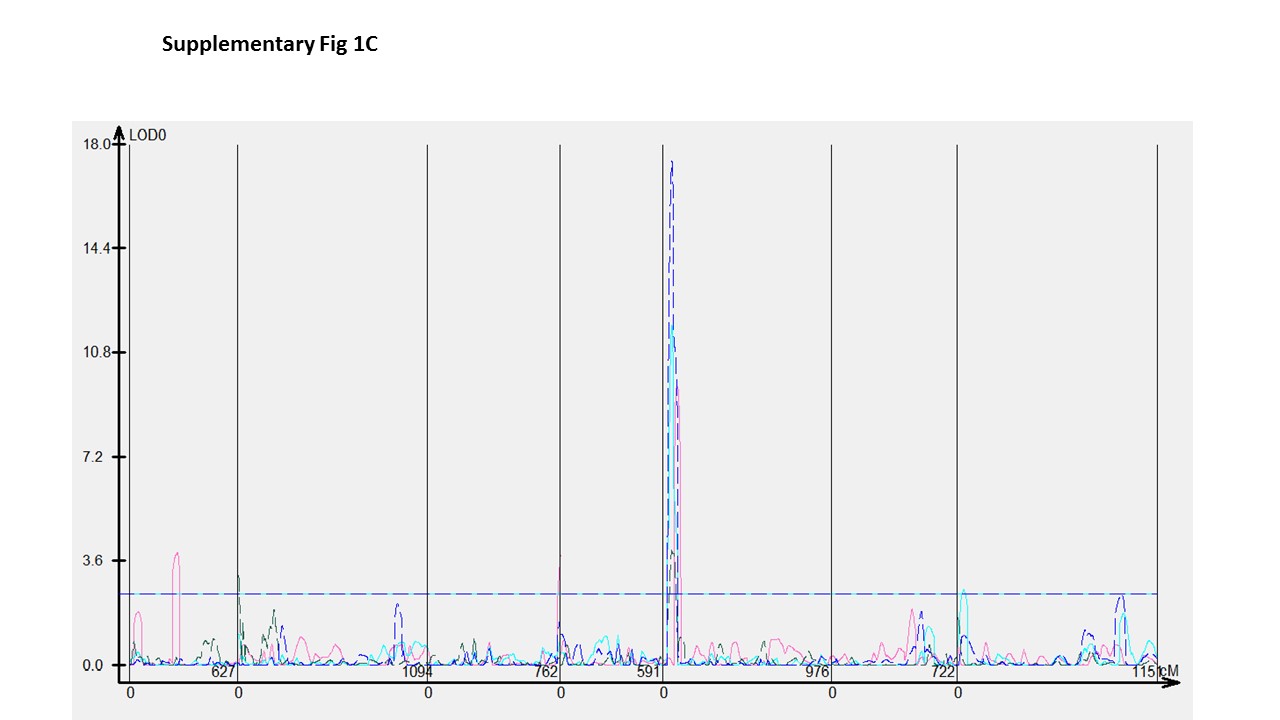

Supplement: FIGURE S1 — LOD trace files for (A) stripe [green and black = WUR greenhouse rep 1 and 2, dark blue, red, and black dash = Mexico and Ecuador field sites and light blue = P. striiformis f. sp. pseudo-hordei], (B)leaf [pink = greenhouse PBI Cobbitty, dark blue dash = Field_PBICobbitty_2018rep1, light blue = Field_PBICobbitty_2018rep2, black dashed = FieldPBICobbitty_2016] and (C) stem rust [red dashed = greenhouse PBI Cobbitty] resistance QTLs identified in the Pompadour × Biosaline-19 RIL population. [file Data_Sheet_1.zip › Image 1C.JPEG]
